# Effects of Dietary Pretreatment with All-*trans* Lycopene on Lipopolysaccharide-Induced Jejunal Inflammation: A Multi-Pathway Phenomenon

**DOI:** 10.3390/foods14050794

**Published:** 2025-02-26

**Authors:** Daolin Mou, Dajiang Ding, Junning Pu, Pan Zhou, Enming Cao, Xueyan Zhang, Junrong Lan, Lu Ye, Wanxue Wen

**Affiliations:** 1 College of Bioengineering, Sichuan University of Science and Engineering, Zigong 643000, China; 2 Institute of Neuroscience, Center for Excellence in Brain Science and Intelligence Technology, State Key Laboratory of Neuroscience, Chinese Academy of Sciences, Shanghai 200031, China; 3 Key Laboratory for Animal Disease-Resistance Nutrition of China Ministry of Education, Institute of Animal Nutrition, Sichuan Agricultural University, Chengdu 611130, China; 4 School of Life Science and Engineering, Southwest University of Science and Technology, Mianyang 621010, China

**Keywords:** lycopene, LPS, intestinal inflammation, antioxidant capacity, microbiome and metabolomics, TLR4/NF-κB

## Abstract

This study was conducted to investigate the effects and mechanisms of all-*trans* lycopene on intestinal health by establishing lipopolysaccharide-induced (LPS-induced) jejunal inflammation model. Dietary lycopene supplementation enhanced serum and jejunum antioxidant capacity. Lycopene significantly reduced LPS-induced upregulation of toll-like receptor-4 (TLR-4) and nuclear factor kappa-B (NF-κB), suggesting that lycopene reduced the activation of TLR-4/NF-κB signaling pathway induced by LPS challenge, and further protected mice from LPS induced jejunal inflammation. Furthermore, lycopene increased jejunal zonula occludens-1 (ZO-1) protein expression that was reduced by LPS challenge, and increased abundance of *Rikenella*, *Lachnospiraceae_NK4A136_group* and *Mucispirillum* potentially associated with reducing gut inflammation. Overall, these results showed that pretreatment with lycopene can improve jejunal inflammation and ensure intestinal health in mice by improving antioxidant capacity, intestinal barrier function, microorganisms potentially associated with anti-inflammatory effects and reducing the activation of TLR-4/NF-κB signaling pathway by LPS. We provided a new insight into lycopene prevented LPS-induced jejunal inflammation by corresponding alterations in serum metabolites and gut microbiota, improving antioxidant capacity and regulating the TLR-4/NF-κB signaling pathway in mice.

## 1. Introduction

The small intestine facilitates nutrients absorption, and also acts as an innate barrier against intestinal pathogens [1,2,3]. As such, the mammalian intestine has evolved a strong immune capacity to maintain a balance between tolerance to innocuous material and strong immunity to harmful ones [4]. However, the invasion of pathogenic microorganisms, imbalance in gut bacteria, reduce intestinal barrier function, and increase in serum LPS concentrations, etc., all contribute to inflammatory bowel disease (IBD) [5,6,7]. In addition, intestinal redox homeostasis is a prerequisite for maintaining its function, while increasing the reactive oxygen species (ROS) can induce oxidative stress and in turn leads to increase in inflammatory cytokines [8]. Therefore, maintenance redox homeostasis is the basis for effective immunity and health of intestinal tract [1,9]. Natural antioxidant compounds can scavenge ROS and increase antioxidant capacity and may have a role in IBD treatment, especially lycopene [10,11].

Lycopene, a fat-soluble carotenoid, is mainly extracted from tomato, papaya, guava and other plants [12]. Lycopene has been reported to improve antioxidant capacity of the body by scavenging ROS, and has anti-inflammatory ability [13,14,15]. Current studies on the effects of lycopene on intestinal inflammation have primarily utilized the mouse colitis model induced by dextran sulfate sodium (DSS), with a focus on investigating intestinal barrier function [11,16,17]. It is worth noting that the disruption of intestinal barrier function leads to the invasion of LPS, which may be responsible for intestinal inflammation. Therefore, studies on the pre-colitis model have mainly found that lycopene can mitigate intestinal inflammation through enhancing intestinal barrier function. In addition, although studies in other animals and experimental models have also found that lycopene can reduce intestinal inflammation, the effects of lycopene on gut health and the underlying mechanisms require more in-depth investigation [18,19,20,21,22,23,24,25,26].

Therefore, in this study, we established an LPS-induced jejunal inflammation model and combined microbiome and metabolomic methods to deeply investigate the effects and mechanisms of lycopene on intestinal health. We provided the first evidence that lycopene prevented LPS-induced jejunal inflammation by modulating serum metabolomics and gut microbiota, enhancing antioxidant capacity, and regulating the TLR-4/NF-κB signaling pathway in mice.

## 2. Materials and Methods

### 2.1. Animals

Animal protocols were approved by the Animal Care and Use Committee of Sichuan Agricultural University (license number: CD-SYXK-2017-015). The trial was carried out according to the Guide for the Care and Use of Laboratory Animals prepared by the Institutional Animal Care and Use Committee of Sichuan Agricultural University. Forty-eight Bal b/c mice (male, 4 weeks) were purchased from GemPharmatech Co., Ltd. (Chengdu, China) and they were housed in individual plastic cages under the conditions of controlled room temperature (22 ± 2 °C) and 12 h light-dark cycle. All mice were randomly divided into two groups (*n* = 24/group): control group (CON group, a basal diet from Chengdu Dossy Experimental Animal Co., Ltd., Chengdu, China) and Lyc group (the basal diet supplemented with 300 mg.kg^−1^ lycopene (HPLC: ≥98%, Xi’an Xiaocao Plant Technology Co., Ltd., Xi’an, China)), the dose of lycopene supplementation was determined according to the previous studies [13,14,15]. The mice had access to water and their respective diets ad libitum for 6 weeks. The feed intake and body weight were measured and presented in Figure S1.

### 2.2. Escherichia coli Lipopolysaccharide Challenge

**Figure 1:**
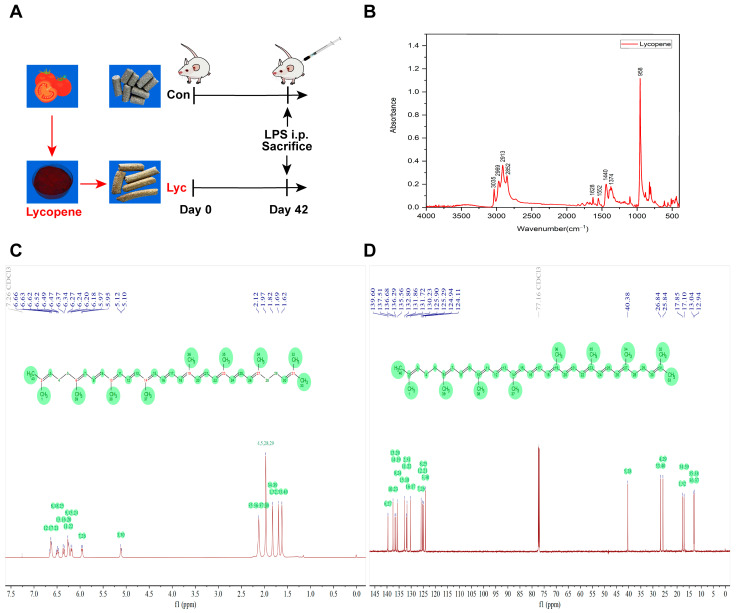
FTIR and NMR spectra of lycopene. (**A**) The procedure of experimental design; (**B**) FTIR spectrum analysis; (**C**) NMR spectra of lycopene of 1H NMR; (**D**) NMR spectra of lycopene of 13C NMR. FTIR, fourier transform infrared spectroscopy; NMR, nuclear magnetic resonance; LPS, lipopolysaccharide.

After six weeks of treatment, LPS challenge experiment was conducted on the end day of the experiment, which was divided into four groups: CON, CON + LPS, Lyc and Lyc + LPS, with twelve replicates in each group, and one mouse in each replicate. The detailed experimental schedule was demonstrated in Figure 1A. CON + LPS and Lyc + LPS groups were injected intraperitoneally with 0.6 μg.g^−1^ body weight LPS (*Escherichia coli* L2880, Sigma, St Louis, MO, USA) on the day of LPS challenge experiment, and the CON and Lyc groups were injected intraperitoneally with an equivalent amount of sterile saline. The LPS dose was based on our previous studies and preliminary experiments [1,3]. All mice were sacrificed 6 h after LPS injection. The serum, jejunum and colonic digesta samples of Bal b/c mice were collected for further analysis.

### 2.3. FTIR Analysis

The lycopene was analyzed by ATR-FTIR with FTIR (Fourier Transform Infrared Spectroscopy) spectrometer (IRAffinity-1S, Shimadzu Corporation, Tokyo, Japan). Lycopene samples were measured under the following conditions: test resolution of 2 cm^−1^, 32 scans, and test range between 600 and 4000 cm^−1^.

### 2.4. NMR Analysis

^1^H NMR and ^13^C NMR spectra of lycopene were determined on a Bruker AVANCE NEO 600M (Bruker Corporation, Billerica, MA, USA) spectrometer. Briefly, lycopene samples were dissolved in deuterated chloroform (CDCl_3_), tetramethylsilane was used as an internal standard [27]. The results were analyzed by MestReNova (Version 14.0.0, Mestrelab Research S.L., A Coruña, Spain) software.

### 2.5. LC-MS/MS Profiling of Serum Untargeted Metabolomics

Untargeted Metabolomics profiling was performed using liquid chromatography mass spectrometry (LC-MS) on serum samples. Prechilled 80% methanol was used to resuspend the samples by vortex. After centrifugation, the supernatant was diluted by water to a solution containing 53% methanol and performed for the LC-MS/MS analysis. Subsequently, the Vanquish UHPLC system (ThermoFisher Scientific, Bremen, Germany) equipped with Q Exactive^TM^ HF mass spectrometer (ThermoFisher Scientific, Bremen, Germany) was used for the LC-MS/MS analyses. 0.1% formic acid in water (A) and methanol (B) were used to develop a gradient elution. The samples were fractionated with a Hypersil Goldcolumn (1.9 μm, 2.1 × 100 mm). The chemical composition (from the positive and negative ion modes) was assessed by the Q Exactive^TM^ HF mass spectrometer. Finally, the Compound Discoverer 3.3 processed the raw data files from LC-MS/MS analyses.

### 2.6. Enzyme Activity Assay

Serum or jejunum samples were prepared and measured as previously described [28,29]. Reduced glutathione (GSH, Cat. No. A006-2-1), oxidized glutathione (GSSG, Cat. No. A061-1-2), total antioxidant capacity (T-AOC, Cat. No. A015-2-1), total superoxide dismutase (T-SOD, Cat. No. A001-1-1), catalase (CAT, Cat. No. A007-1-1), glutathione peroxidase (GSH-Px, Cat. No. A005-1-2), and malondialdehyde (MDA, Cat. No. A003-1-1) were measured in a 96-well microplate by using commercial kits purchased from Nanjing Institute of Jiancheng Biological Engineering (Nanjing, China) according to the manufacturer’s instructions.

### 2.7. Serum TNF-α and IL-6 Analysis

Serum tumor necrosis factor-α (TNF-α, Cat. No. YJ002095-4) and interleukin 6 (IL-6, Cat. No. YJ063159-4) concentrations were evaluated by using the commercial ELISA kits purchased from Shanghai Enzyme-linked Biotechnology Co., Ltd. (Shanghai, China). The levels of TNF-α, and IL-6 were calculated from the standard curve and were expressed as picograms per milliliter. Coefficient of variation intraassay and interassay were less than 10%.

### 2.8. Real-Time Quantitative PCR

Total RNAs from jejunum were extracted by E.Z.N.A.^®^ Total RNA Kit I reagent (Omega Bio-Tek, Norcross, GA, USA). The total RNA was reversely transcribed into cDNA by using a HiScript^®^ III RT SuperMix for qPCR (+gDNA wiper) kit (Vazyme, Nanjing, China). RT-qPCR was used to relatively quantify mRNA by a ChamQ Universal SYBR qPCR Master Mix kit (Vazyme, Nanjing, China). Primers for the target genes were presented in Table S1. The PCR cycling conditions were 40 cycles of 95 °C for 15 s and 60 °C for 60 s. The 2^−ΔΔCt^ method was used to calculate the relative gene expressions and normalized to GAPDH mRNA.

### 2.9. Immunohistochemistry

Immunohistochemistry of jejunal TLR-4 and NF-κB p65 protein was analysis as previously described [1]. Briefly, the jejunal samples from mice were collected, stored in 4% paraformaldehyde (Beyotime, Shanghai, China), embedded in paraffin, and sliced into 4 µm using a pathology microtome (Leica, Wetzlar, Germany). After being deparaffinized, the sections were washed with graded alcohol, rehydrated in phosphate-buffered saline (PBS), boiled in citrate buffer (pH 6.0, 10 mmol.L^−1^), and then blocked with 3% BSA for 30 min. Subsequently, sections were incubated with primary antibody of mouse monoclonal anti-TLR4 (1:100, ab22048, Abcam, Cambridge, UK) and rabbit anti-NF-κB p65 (D14E12) (1:500, #8242, Cell Signaling Technology, Danvers, MA, USA) overnight at 4 °C. Then, the sections were incubated with specific secondary antibodies and 3,3-diamino benzidine tetrahydrochloride (DAB) to visualize immune complexes. Images were captured by BA200 Digital microscope (Motic, Xiamen, China). The quantitative histomorphometry assessments were assessed by ImageJ (Version 1.46) image processing and analysis software (NIH, Bethesda, MD, USA).

### 2.10. NF-κB p65 Activity Assay

Nuclear extracts were isolated from jejunum tissues using a commercially available nuclear extract kit (#ab113474, Abcam, Cambridge, UK). The DNA binding activity of NF-κB (p65) in the nuclear fraction was evaluated by the NF-κB p65 Transcription Factor Assay Kit (#ab133112, Abcam, Cambridge, UK).

### 2.11. Immunofluorescence

Primary antibodies of TNF-α (#CL488-60291, Proteintech, Wuhan, China) and ZO-1 (#339100, Invitrogen, Carlsbad, CA, USA) were used for paraffin or frozen section samples, and the immunofluorescence experimental procedures refer to instructions of the corresponding manufacturer. Briefly, jejunum sections were fixed with 4% paraformaldehyde (Beyotime, Shanghai, China), blocked with 1% BSA for 1h, and incubated with primary antibodies (TNF-α, 1:50; ZO-1, 1:500) at 4 °C overnight, followed by being incubated with Alexa Fluor 488 secondary antibodies (1:1000) and 1 mg.mL^−1^ DAPI (#8961, CST, Danvers, MA, USA) in darkness. Finally, the sections were mounted in mounting medium (Southern Biotech, #0100-01) and placed under a FV3000 confocal microscope (Olympus, Hachioji-shi, Japan) to observe and take pictures.

### 2.12. Microbial Analyses

Mouse gut microbiome was analyzed at Novogene Bioinformatics Technology (Beijing, China) based on the full-length 16S rRNA amplicon sequencing. Briefly, the CTAB (cetyltrimethylammonium bromide) method was used to obtain total genome DNA. The TransStart^®^ FastPfu DNA Polymerase (TransGen Biotech, Beijing, China) was used to amplify the 16S rRNA genes of distinct regions. PCR products was mixed with 1× loading buffer and purified with QIAquick@ Gel Extraction Kit (QIAGEN, Shanghai, China). And finally, the SMRTbellTM Template Prep Kit (PacBio, Menlo Park, CA, USA) was used to generate the sequencing libraries for the following sequencing. The sequences were clustered into Operational Taxonomic Units (OTUs) based on 97% sequence similarity by Uparse software (v7.0.1001), and representative sequences of OTUs (the highest frequency in OTUs) are selected [30]. Alpha diversity (Observed-species and Chao1 indices) was calculated using QIIME (Version1.9.1) and the rarefaction curves were drawn using R (Version 2.15.3) software. Beta diversity was calculated by QIIME software. Biomarker discovery responses to Lyc treatment were analyzed using the linear discriminant analysis effect size (LEfSe) with an LDA score of higher than 3.

### 2.13. Statistical Analyses

SPSS 26.0 software (SPSS Inc., Chicago, IL, USA) was used for statistical analysis. Data were obtained via the general linear model (GLM) in the following model: Y_ijk_ = μ + α_i_ + β_j_ + (αβ)_ij_ + ε_ijk_, in which Y is the analyzed variable; μ is the mean; α_i_ is the effect of Lyc (i = 1, or 2); β_j_ is the effect of LPS (j = 1 or 2); (αβ)_ij_ refers to the interaction between Lyc and LPS; and ε_ijk_ represents the error term. Additionally, Tukey’s multiple range test was used to confirm treatment effects and whether there were differences between each group. The Shapiro–Wilk W test and Levene’s test were used to evaluate the normality and homogeneity of variances. Data were presented as means and standard deviation (SD). *p* ˂ 0.05 was considered to be statistically significant between groups. The correlation analysis between the indicators and gut microbiota was performed using Pearson correlation analysis and was completed by Origin 2021 (OriginLab, Northampton, MA, USA). The results were plotted with GraphPad Prism 9.5 software (Graphpad, San Diego, CA, USA).

## 3. Results

### 3.1. FTIR and NMR Spectra of Lycopene

The FTIR spectra of the lycopene were shown in Figure 1B, both exhibited an absorption peak at 3035 cm^−1^, 2969 cm^−1^, 2913 cm^−1^ and 2852 cm^−1^ corresponded to the C-H stretching vibration. The peak at 1628 cm^−1^, 1552 cm^−1^ corresponded to the C=C stretching vibration, and the infrared absorption peak detected at 1440 cm^−1^ and 1374 cm^−1^ corresponded to the C-H bending vibration. Similarly to previous studies, lycopene sample exhibited a strong characteristic absorption peak at approximately 958 cm^−1^, attributed to the presence of *trans*-C-H out-of-plane deformation vibration of lycopene found in tomatoes [31,32,33]. All these absorption peaks correspond to lycopene in the sample, and the strong peak at 958 cm^−1^ is its characteristic peak.

The NMR spectra of lycopene including ^1^H and ^13^C were shown in Figure 1C,D. The identity was confirmed by NMR analysis. ^1^H NMR (*δ*, ppm, 600 MHz, CDCl_3_): *δ* 6.63 (d, J = 10.1 Hz, 4H), 6.49 (t, J = 13.1 Hz, 2H), 6.36 (d, J = 14.9 Hz, 2H), 6.25 (d, J = 13.6 Hz, 4H), 6.19 (d, J = 11.5 Hz, 2H), 5.96 (d, J = 10.9 Hz, 2H), 5.11 (d, J = 7.9 Hz, 2H), 2.12 (s, 8H), 1.97 (s, 12H), 1.82 (s, 6H), 1.69 (s, 6H), 1.62 (s, 6H); ^13^C NMR (*δ*, ppm, 151 MHz, CDCl_3_): δ 139.60, 137.51, 136.68, 136.29, 135.56, 132.80, 131.86, 131.72, 130.23, 125.90, 125.29, 124.94, 124.11, 40.38, 26.84, 25.84, 17.85, 17.10, 13.04, 12.94. All of the peaks indicated all-trans structure and high purity of the lycopene sample [27].

### 3.2. Effect of Lycopene on Metabolomics of Mice Serum

**Figure 2:**
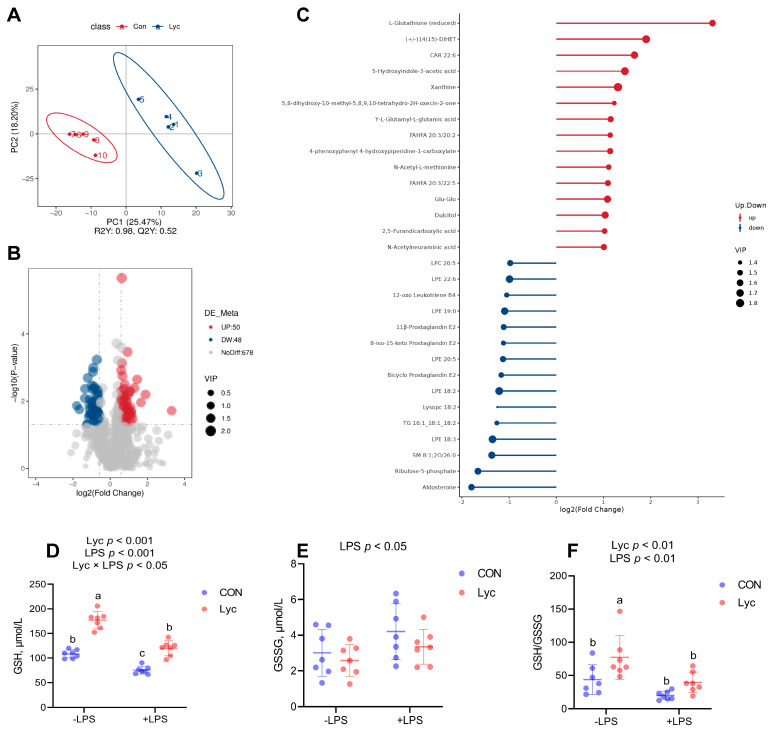
Effect of lycopene on serum metabolomics of mice. (**A**) Partial least squares discriminant analysis (PLS-DA) plot of metabolites (*n* = 5 per group), PLS-DA were performed at metaX; (**B**) Volcano plot of differential metabolites (*n* = 5 per group), volcano plots were used to Sifting relative metabolites by ggplot2 in R language based on log2 (Fold Change) and -log10 (*p*-value); (**C**) Matchstick plot of differential metabolites (top 30) (*n* = 5 per group), VIP > 1 and *p*-value (*t*-test) < 0.05 and fold change ≥ 2 or fold change ≤ 0.5 were identified as differential metabolites. Serum (**D**) GSH and (**E**) GSSG concentrations, (**F**) the ratio of GSH to GSSG (*n* = 7 per group); −LPS, mice not challenged with LPS; +LPS, mice challenged with LPS; Data were expressed as means ± SD; ^a,b^ Columns with variant alphabetical superscript mean significant differences (*p* < 0.05).

As shown in Figure 2, lycopene supplementation significantly affected serum metabolites. The plots of experimental samples from CON and Lyc groups were separated, indicating that the metabolites of the two groups were distinguishable from each other by significant differences, and that there was intragroup aggregation of metabolites in the two groups (Figure 2A). Volcano plot of differential metabolites showed that 50 metabolites were significantly up-regulated and 48 metabolites were significantly down-regulated between the two groups (Figure 2B). Matchstick plot top 15 metabolites for up- and down-regulation, among which up-regulated metabolites of L-glutathione (reduced), (+/−)14(15)-DiHET, 5-hydroxyindole-3-acetic acid and down-regulated metabolites in Aldosterone, Ribulose-5-phosphate had large fold differences and large VIP values (Figure 2C). We further analyzed the concentrations of serum GSH and GSSG because of the large multiplicative changes in L-Glutathione (reduced). It was found that lycopene significantly increased serum GSH concentration and reversed the decrease in GSH concentration caused by LPS challenge (Figure 2D). At the same time, lycopene treatment also increased the GSH/GSSG ratio (Figure 2F).

### 3.3. Effect of Lycopene on the Serum Antioxidant Capacity and Inflammatory Factor Concentrations of Mice

**Figure 3:**
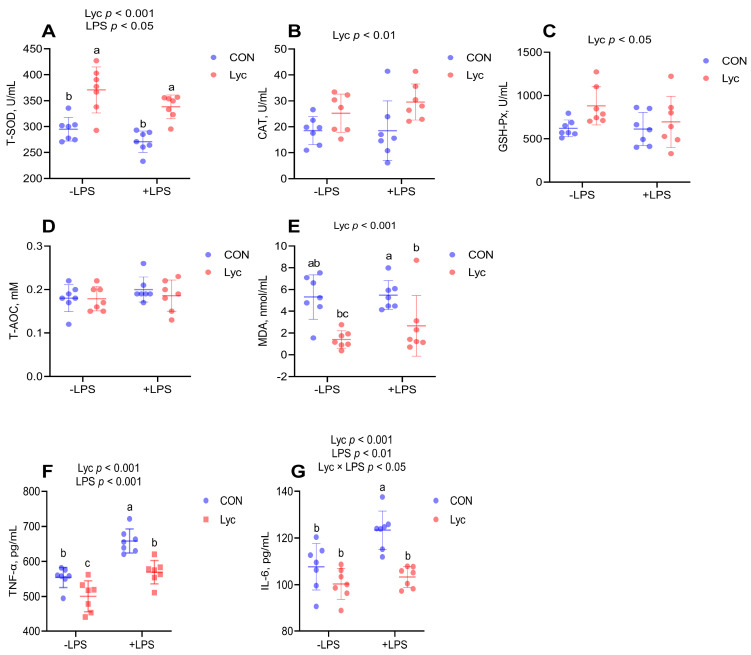
Effect of lycopene on the serum antioxidant capacity and inflammatory factor concentrations of mice. (**A**–**E**) Serum redox status in mice (*n* = 7 per group); (**F**,**G**) Serum inflammatory factors in mice (*n* = 7 per group). −LPS, mice not challenged with LPS; +LPS, mice challenged with LPS. Data were expressed as means ± SD. ^a,b^ Columns with variant alphabetical superscript mean significant differences (*p* < 0.05).

Lycopene supplementation significantly increased serum T-SOD, CAT and GSH-Px activities and decreased serum MDA, TNF-α and IL-6 concentrations in mice (Figure 3). LPS challenge significantly decreased serum T-SOD activity and significantly increased serum TNF-α and IL-6 concentrations (Figure 3). Lycopene treatment significantly decreased serum TNF-α and IL-6 levels increased by LPS challenge (Figure 3F,G). These results indicated that lycopene effectively improved serum antioxidant capacity and reversed the elevation of inflammatory cytokines induced by LPS challenge.

### 3.4. Effect of Lycopene on the Jejunal Antioxidant Capacity of Mice

**Figure 4:**
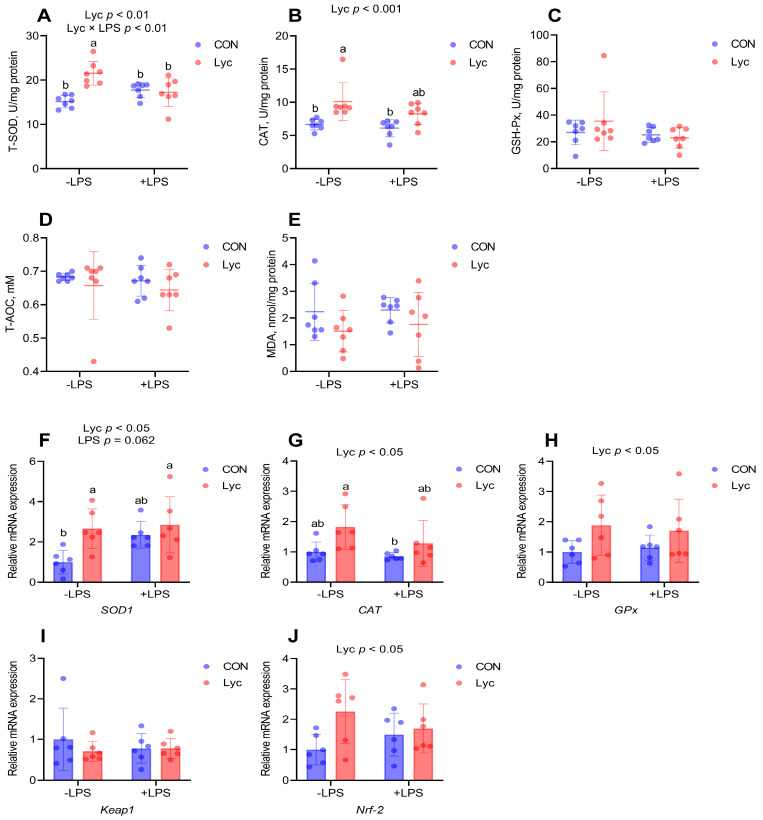
Effect of lycopene on the jejunal antioxidant capacity of mice. (**A**–**E**) Redox state of mice jejunum (*n* = 7 per group); (**F**–**J**) Relative mRNA expression of jejunal redox-related genes were detected by RT-PCR (*n* = 6 per group). −LPS, mice not challenged with LPS; +LPS, mice challenged with LPS; SOD1, superoxide dismutase 1; CAT, catalase; GPx, glutathione peroxidase; Keap1, kelch-like ECH-associated protein l; Nrf-2, nuclear factor erythroid-2 related factor 2. Data were expressed as means ± SD. ^a,b^ Columns with variant alphabetical superscript mean significant differences (*p* < 0.05).

As shown in Figure 4A,B, lycopene supplementation significantly increased jejunal T-SOD and CAT activities. In addition, lycopene treatment significantly affected jejunal *SOD1*, *CAT*, *GPx* and *Nrf-2* mRNA expressions (Figure 4). Compared to the control group, Lyc and Lyc + LPS groups had higher expression of *SOD1* (Figure 4F). Furthermore, Lyc group jejunal *CAT* relative expression level was higher than CON + LPS group (Figure 4G). These results indicated that lycopene improved antioxidant capacity of mice jejunum.

### 3.5. The Potential Mechanism of Lycopene on Inflammation of the Jejunum

**Figure 5:**
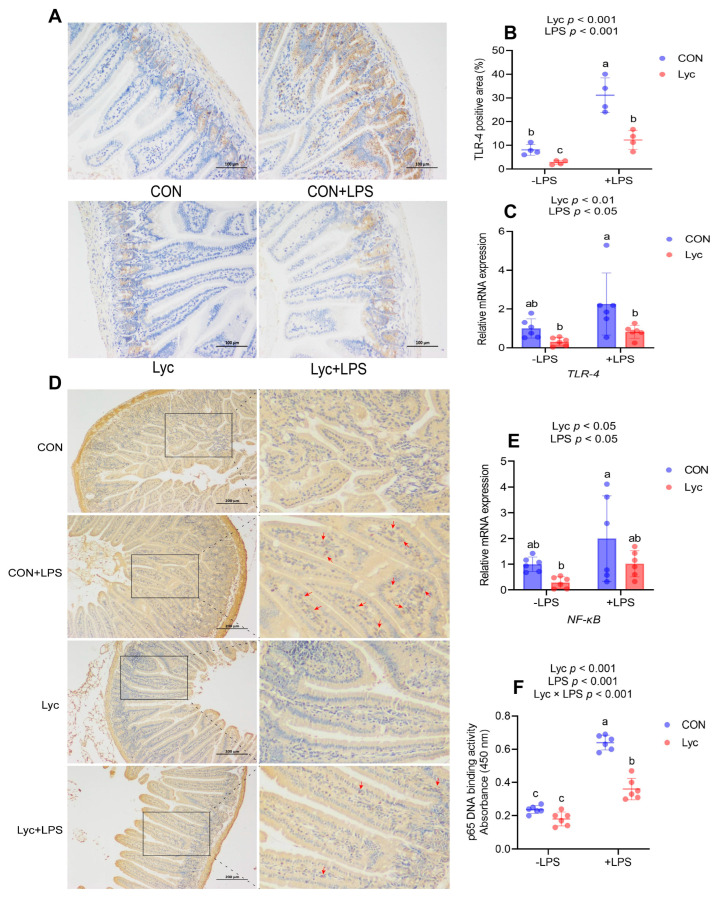
Effect of lycopene on the jejunal TLR-4/NF-κB signaling pathway of mice. (**A**,**B**) IHC staining and quantitative analysis of TLR-4 in mice jejunum. Scale bar, 100 μm. *n* = 4 per group. (**C**) Relative mRNA expression of jejunal *TLR-4* was detected by RT-PCR (*n* = 6 per group). (**D**) IHC staining of NF-κB in mice jejunum. Scale bar, 200 μm. *n* = 4 per group. The red arrows represented the positive cells. (**E**) Relative mRNA expression of jejunal *NF-κB* was detected by RT-PCR (*n* = 6 per group). (**F**) NF-κB p65 activity in mice jejunum (*n* = 6 per group). −LPS, mice not challenged with LPS; +LPS, mice challenged with LPS; TLR-4, toll-like receptor-4; NF-κB, nuclear factor kappa B. Data were expressed as means ± SD. ^a,b^ Columns with variant alphabetical superscript mean significant differences (*p* < 0.05).

To further investigate the potential mechanism of lycopene on jejunal inflammation, we analyzed the TLR-4/NF-κB signaling pathway under the LPS-induced inflammation model. We found that lycopene significantly reduced LPS-induced upregulation of TLR-4 gene and protein expression (Figure 5A–C). In addition, lycopene treatment decreased NF-κB expression level and significantly reduced the increase in NF-κB p65 DNA-binding activity induced by LPS challenge (Figure 5D–F). These results suggested that lycopene treatment reduced the activation of TLR-4/NF-κB pathway induced by LPS challenge.

### 3.6. Effect of Lycopene on the Jejunal Inflammatory Factor and ZO-1 Protein Expression of Mice

**Figure 6:**
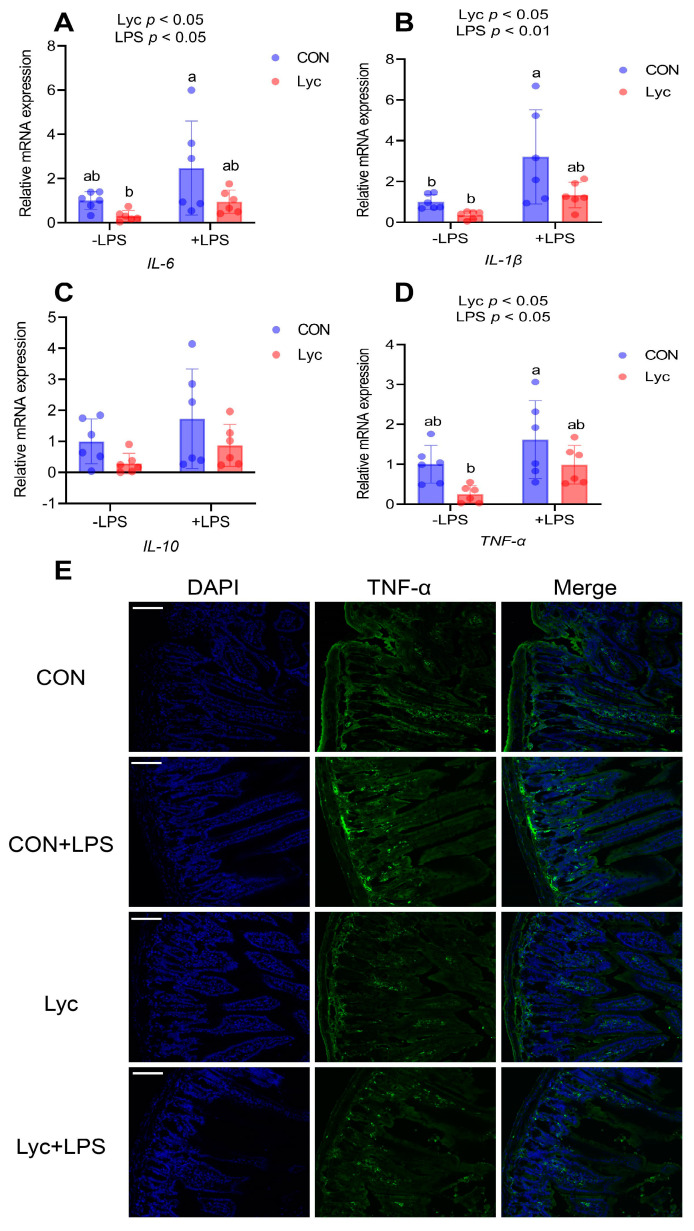
Effect of lycopene on the jejunal inflammatory factor expression of mice. (**A**–**D**) Relative mRNA expression of jejunal *IL-6*, *IL-1β*, *IL-10* and *TNF-α* were detected by RT-PCR (*n* = 6 per group). (**E**) Immunofluorescence staining of TNF-α in mice jejunum. Scale bar, 100 μm. *n* = 4 per group. −LPS, mice not challenged with LPS; +LPS, mice challenged with LPS; IL-6, interleukin 6; IL-1β, interleukin 1β; IL-10, interleukin 10; TNF-α, tumor necrosis factor α. Data were expressed as means ± SD. ^a,b^ Columns with variant alphabetical superscript mean significant differences (*p* < 0.05).

**Figure 7:**
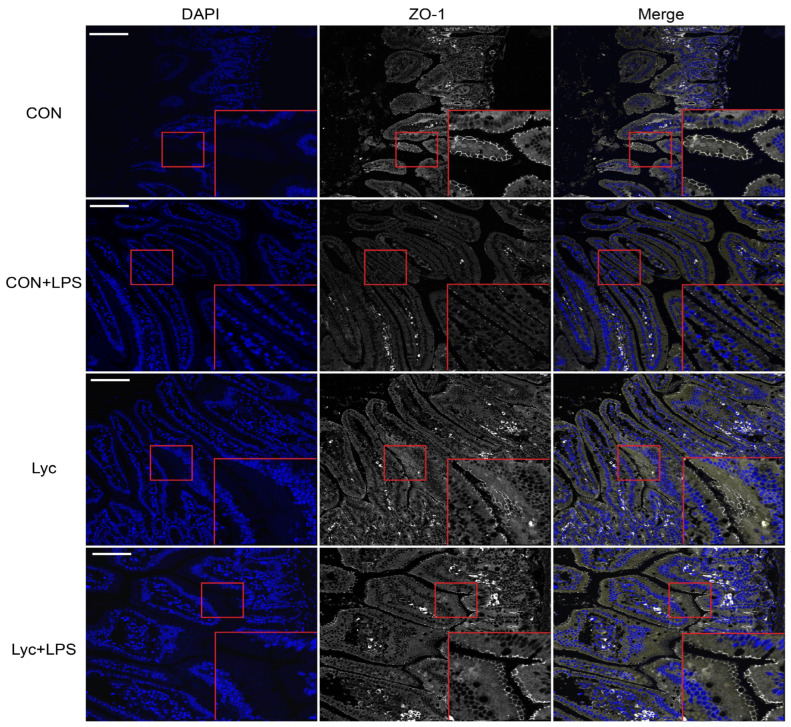
Effect of lycopene on the jejunal ZO-1 protein expression of mice. Immunofluorescence staining of ZO-1 in mice jejunum. Scale bar, 100 μm. Red box, the selected area of zooms in. *n* = 4 per group.

Lycopene supplementation significantly affected the gene expressions of *IL-6*, *IL-1β* and *TNF-α* in jejunum (Figure 6). LPS challenge significantly upregulated jejunal inflammatory factor expression (Figure 6). Compared to CON + LPS group, the gene expression levels of jejunal *IL-6*, *IL-1β* and *TNF-α* were lower in the Lyc group (Figure 6). Lycopene treatment reduced jejunal TNF-α (Figure 6E) protein expression that were elevated by LPS challenge. In addition, lycopene treatment increased jejunal ZO-1 (Figure 7) protein expression that were reduced by LPS challenge. These results indicated that lycopene decreased jejunal inflammation and improved the intestinal barrier function in mice.

### 3.7. Effect of Lycopene Supplementation on Gut Microbiota in Mice

**Figure 8:**
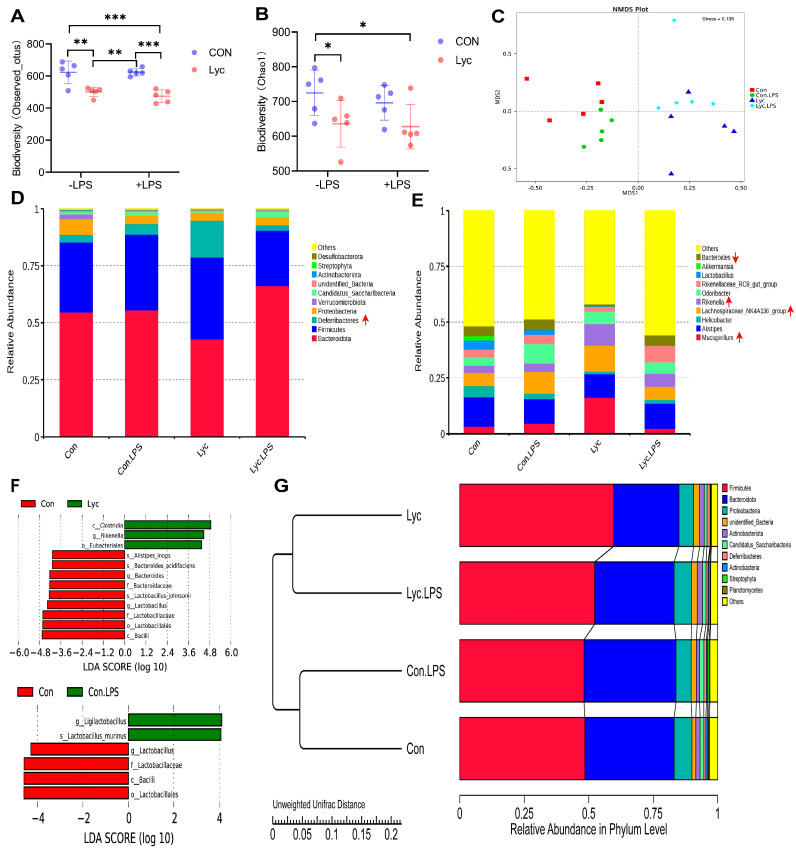
Effect of lycopene on gut microbiota in mice. (**A**,**B**) Bacterial alpha diversity based on Observed_otus and Chao1 index. Data were expressed as means ± SD (*n* = 5 per group). *** *p* < 0.001, ** *p* < 0.01, * *p* < 0.05. (**C**) Beta diversity shown in the scatterplot from non-metric multi-dimensional scaling (NMDS), the distance between points indicates the degree of difference, and samples from the same group are represented using the same color. (**D**,**E**) Shifts in intestinal bacterial taxonomic compositions at phylum (**D**) and genus (**E**) levels. Upward and downward arrows show that the relative abundance of the corresponding taxonomic levels significantly increased and decreased compared with Lyc and CON groups. The significantly differentially abundant bacterial taxonomic levels were identified by Metastats analysis. *n* = 5 per group. Linear discriminant analysis coupled with effect size (LEfSe) (**F**) and unweighted pair-group method with arithmetic mean (UPGMA) (**G**) analysis of the microbiota composition. *n* = 5 per group.

We compared bacterial diversity (Observed_otus and Chao1 index) indices for alpha diversity. The Observed_otus and Chao1 indices in the Lyc group were significantly lower than CON group (*p* < 0.05) (Figure 8A,B). As shown in Figure 8C, the beta diversity shown in the scatterplot from the NMDS (non-metric multi-dimensional scaling), the distribution of microbiota in the CON and Lyc groups were distinctly and separately clustered, indicating that Lyc supplementation significantly changed the structure of bacteria.

The abundance of gut microbiota in the top 10 phyla and genus were presented in Figure 8D,E. The significantly differentially abundant microbial species between CON group and Lyc group were analyzed by Metastats analysis. In terms of phyla level, compared with CON group, Lyc significantly increased the abundance of *Deferribacteres* (Figure 8D). At genus level, lycopene treatment significantly increased abundance of *Rikenella*, *Lachnospiraceae_NK4A136_group* and *Mucispirillum* but decreased the abundance of *Bacteroides* compared with the control (Figure 8E).

Microbial compositions between CON and Lyc (or CON + LPS) were further analyzed using the linear discriminant analysis coupled with effect size (LEfSe) (Figure 8F). In genus level, *Rikenella* was higher in Lyc group compared to control group, to the contrary, *Bacteroides* and *Lactobacillus* were higher in CON group. Moreover, *Ligilactobacillus* was higher in CON + LPS compared to CON, whereas *Lactobacillus* was lower in CON + LPS compared to CON (Figure 8F). In addition, the UPGMA (unweighted pair-group method with arithmetic mean) analysis conducted at the phylum level revealed two primary branches within phylogenetic tree, and the species similarity was highest in CON and CON + LPS groups (or Lyc and Lyc + LPS groups), suggesting that Lyc could modulate the microbial composition (Figure 8G).

### 3.8. Correlation Analysis

**Figure 9:**
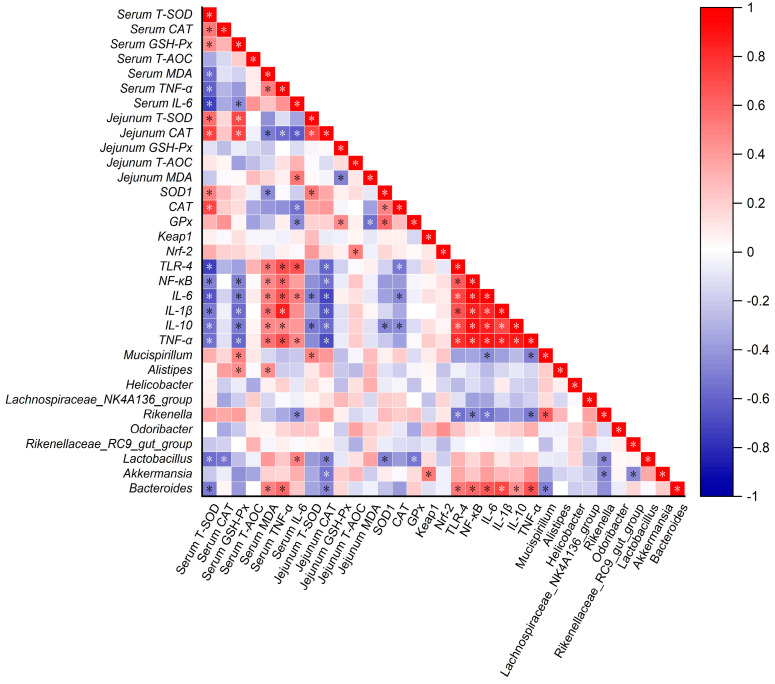
Correlation analysis between the measured relevant indicators and the gut microbiota. The color of red represents a positive correlation, blue represents a negative correlation, “*” indicates a significant correlation ship, *p* < 0.05.

To estimate the potential correlation between the measured relevant indicators and the gut microbiota, the Pearson correlation analysis was performed (Figure 9). As expected, nearly all the inflammation-related indicators were positively correlated with each other, and almost all antioxidant related indicators were negatively correlated with inflammation-related indicators. Moreover, the antioxidant-related indicators were basically positively correlated in serum, jejunal tissue and gene expression. Inflammation related indicators (*TLR-4*, *NF-κB*, *IL-6*, *IL-1β*, *IL-10* and *TNF-α*) were significantly negatively correlated with serum T-SOD and jejunum CAT activities, while exhibited significantly positively correlations with serum MDA and TNF-α concentrations. The results of the correlation analyses between gut microbiota at the genus level (top 10) and other indicators found that *Bacteroides* was significantly positively correlations with inflammation related indicators (*TLR-4*, *NF-κB*, *IL-6*, *IL-1β*, *IL-10* and *TNF-α*), serum MDA and serum TNF-α, while significantly negative correlations with serum T-SOD, jejunum CAT and *Mucispirillum*. In addition, *Mucispirillum* was significantly positively correlations with serum GSH-Px, jejunum T-SOD and *Rikenella*, but was negatively correlated with jejunal *IL-6* and *TNF-α*. In addition, *Rikenella* had significantly negative correlations with inflammation-related indicators (serum IL-6, *TLR-4*, *NF-κB*, *TNF-α* and *IL-6*).

## 4. Discussion

Maintaining the balance of intestinal redox and inflammation is of utmost importance to ensure intestinal health. Although the ability of lycopene to reduce DDS-induced inflammation by improving intestinal barrier function was demonstrated in the mouse model of colitis, the effects and mechanisms of lycopene on intestinal antioxidant capacity, inflammation, and gut microbiota need to be further investigated [11,16,17]. In particular, in-depth investigation of lycopene regulation of intestinal TLR-4/NF-κB signaling pathway through direct establishment of LPS-induced intestinal inflammation has not been reported. In this study, the underlying mechanisms of lycopene on jejunal inflammation was explored by metabolomics, antioxidant capacity, inflammatory pathway, ZO-1 protein and gut microbiome analysis in mice. Our results revealed that lycopene improved antioxidant capacity of serum and jejunum and inhibited TLR-4/NF-κB signaling activation, indicating that inhibition of jejunal inflammation by lycopene.

As one of the strongest antioxidants in nature, lycopene can effectively scavenge ROS [34,35]. Moreover, in vivo lycopene was found to increase the activity of antioxidant enzymes such as SOD, CAT, etc. [13,36]. In addition, lycopene had been found to protect the intestinal epithelium from oxidative damage by modulating the Keap1/Nrf2 signaling pathway under deoxynivalenol exposure [24]. Therefore, this study systematically analyzed the influences of lycopene on the antioxidant ability of serum and mice jejunum. In the current study, metabolomics data showed that lycopene treatment could upregulate the serum metabolite of L-Glutathione (reduced), so we further analyzed the serum GSH and GSSG levels of mice, and the results also found that lycopene increased GSH concentration and the GSH/GSSG ratio. Analysis of other redox indicators of serum also found that lycopene supplementation significantly increased serum T-SOD, CAT and GSH-Px activities and decreased serum MDA concentrations in mice. In addition, lycopene supplementation significantly increased T-SOD, CAT activities and related gene expression in jejunum. SOD, CAT, and GSH-Px exert tremendous protective roles in intestinal epithelium against inflammation [9]. These results suggested that lycopene improved antioxidant capacity in mice, providing a prerequisite for modulating jejunal inflammation.

Previous studies have demonstrated the ability of lycopene to reduce symptoms of DSS-induced colitis [11,16,17], and lycopene decreases the levels of pro-inflammatory cytokines have been reported to be logical targets for IBD therapy [11,37]. LPS is a potent inducer of inflammation and has been used in experiments to mimic bacterial infections in animals [1,6]. LPS stimulation activates the immune system and enhances the secretion of pro-inflammatory cytokines, such as TNF-α and IL-6, through the TLR-4/NF-κB signaling pathway. This pathway plays a crucial role in modulating intestinal inflammatory responses [1,3]. In addition, previous studies found that LPS could induce ROS production when activating TLR4-NF-κB signal [38,39]. Moreover, ROS production by inflammatory cells can induce oxidative stress [8], and ROS can enhance LPS-induced inflammatory cytokines production [40], while antioxidant substances may improve the release of LPS-induced pro-inflammatory factors by clearing ROS. Hence, to further investigate the underlying mechanism of lycopene on jejunal inflammation, we analyzed the TLR-4/NF-κB signaling pathway under the LPS-induced inflammation model. Here, we showed that the LPS challenge significantly increased the expression of TLR-4 at the gene and protein levels, the activity of NF-κB p65 DNA binding, and the concentrations of serum TNF-α and IL-6. On the contrary, pretreatment with lycopene significantly reduced activation of TLR-4/NF-κB signaling pathway. On the other hand, we also analyzed the expression level of the intestinal tight junctions (TJs) protein ZO-1. Upregulation of TJs expression implies the reduced risk of intestinal inflammatory diseases [3]. Numerous pieces of evidence verify that lycopene can upregulation of intestinal ZO-1 expression [11,41]. Similarly, our results found that lycopene treatment increased jejunal ZO-1 protein expression that were reduced by LPS challenge. In the present study, our data suggested that pretreatment with lycopene could improve jejunal inflammation and ensured intestinal health by improving antioxidant capacity, intestinal barrier function, and reducing activation of the TLR-4/NF-κB pathway induced by LPS.

Imbalance in gut bacteria and dysregulated immune response to gut microbes are central to IBD [7]. The maintenance of a gut ecosystem involves complex interactions between the host, resident microbial communities, and invading pathogens [42]. However, the link between the gut bacteria and intestinal inflammation is not well understood, and through the modulation of gut microbes, which, in turn, reduces intestinal inflammation, remains to be further investigated. Specific evidence for the modulation of gut microbes by lycopene is lacking. In the present study, lycopene treatment significantly increased the relative abundance of *Rikenella*, *Mucispirillum* and *Lachnospiraceae_NK4A136_group*, while decreasing the abundance of *Bacteroides* compared to the control group. *Rikenella* appears to be a crucial member of the gut bacteria and a potential probiotic that may be necessary to reduce intestinal inflammation [43,44]. Evidence indicates that in human patients with IBD, the abundance of *Rikenella* is significantly reduced compared to healthy individuals [45], and *Rikenella* can strengthen the intestinal barrier [46]. As a potential probiotic, *Lachnospiraceae_NK4A136_group* is negatively correlated with chronic inflammation [47,48], and has a potential anti-colitis activity [49,50]. *Mucispirillum schaedleri*, a member of *Deferribacteres* phylum presented in the gut microbiota of mice and humans, protects animals against colitis caused by *S. Typhimurium* [51]. To the contrary, *Bacteroides* are associated with colitis in IBD [52]. In addition, it is worth noting that the results of our correlation analysis also found *Bacteroides* was significantly positively correlated with inflammation related indicators; *Mucispirillum* was signally positively correlated with serum GSH-Px, jejunum T-SOD, but was significantly negatively correlated with IL-6 and TNF-α. Taken together, these data suggested that lycopene increased the abundance of bacteria (*Rikenella*, *Lachnospiraceae_NK4A136_group* and *Mucispirillum*) that are potentially associated with reducing gut inflammation, but reduced the abundance of bacteria (*Bacteroides*) that may promote intestinal inflammation. However, whether these changes in microbial abundance will further affect physiological functions remains to be further investigated.

## 5. Conclusions

Dietary lycopene supplementation improved serum and jejunal antioxidant capacity while reducing the jejunal inflammation level of mice by suppressing TLR-4/NF-κB pathways, and further protected mice from LPS induced jejunal inflammation. We also found that lycopene increased jejunal ZO-1 protein expression that was reduced by LPS challenge, and increased bacteria (*Rikenella*, *Lachnospiraceae_NK4A136_group* and *Mucispirillum*) potentially associated with reducing gut inflammation. Therefore, mice pretreatment with lycopene could improve jejunal inflammation and ensure intestinal health by improving antioxidant capacity, intestinal barrier function, microorganisms potentially associated with anti-inflammatory effects and reducing activation of TLR-4/NF-κB signaling pathway induced by LPS. This study indicated that the role of lycopene in improving jejunal inflammation, and the mechanism by which lycopene modulates LPS-induced jejunal inflammation was elucidated.

## Data Availability

The original contributions presented in this study are included in the article/Supplementary Materials. Further inquiries can be directed to the corresponding author.

## Supplementary Materials

The following supporting information can be downloaded at: https://www.mdpi.com/article/10.3390/foods14050794/s1, Figure S1: Effects of lycopene on body weight and feed intake in mice; Table S1: Primer sequences of the target and reference genes.
