# Supplementary material for: Effects of Dietary Pretreatment with All-trans Lycopene on Lipopolysaccharide-Induced Jejunal Inflammation: A Multi-Pathway Phenomenon
Source: Foods. 2025 Feb 26;14(5):794. doi: 10.3390/foods14050794 (PMC11898642; doi:10.3390/foods14050794)
Supplement: Supplementary file 1 [file foods-14-00794-s001.zip › foods-3482914-supplementary.pdf]

Supplementary data

Supplemental Figure

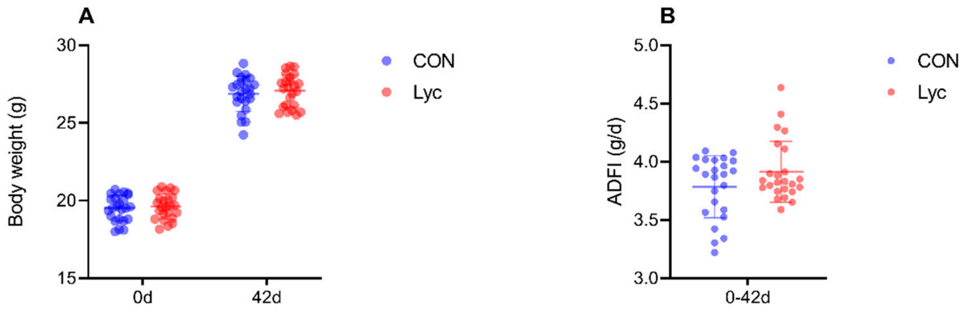

Supplemental Figure S1. Effects of lycopene on body weight and feed intake in mice. Data were expressed as means ± SD (n = 24 per group). ADFI, average daily feed intake.

Supplemental table

Supplemental Table S1. Primer sequences of the target and reference genes

Genes	Primer	Sequence (5'→3')
SOD1	Forward	5'-GGAACCATCCACTTCGAGCA-3'
	Reverse	5'-CCCATGCTGGCCTTCAGTTA-3'
CAT	Forward	5'-GCGGATTCCTGAGAGAGTGG-3'
	Reverse	5'-TGGAGAACCGAACGGCAATA-3'
GPx	Forward	5'-CTCAAGTACGTCCGACCTGG-3'
	Reverse	5'-TAAAGAGCGGGTGAGCCTTC-3'
Nrf-2	Forward	5'-AAAGCACAGCCAGCACATTC-3'
	Reverse	5'-TGGGATTCACGCATAGGAGC-3'
Keap1	Forward	5'-CTCAACCGCTTGCTGTATGC-3'
	Reverse	5'-TTCAACTGGTCCTGCCCATC-3'
TLR-4	Forward	5'-ATGGCATGGCTTACACCACC-3'
	Reverse	5'-GAGGCCAATTTTGTCTCCACA-3'
NF-κB1	Forward	5'-ATGGCAGACGATGATCCCTAC-3'
	Reverse	5'-TGTTGACAGTGGTATTTCTGGTG-3'
TNF-α	Forward	5'-CATCTTCTCAAAATTCGAGTGACAA-3'
	Reverse	5'-TGGGAGTAGACAAGGTACAACCC-3'
IL-1β	Forward	5'-GAAATGCCACCTTTTGACAGTG-3'
	Reverse	5'-TGGATGCTCTCATCAGGACAG-3'
IL-6	Forward	5'-TAGTCCTTCCTACCCCAATTTCC-3'
	Reverse	5'-TTGGTCCTTAGCCACTCCTTC-3'
IL-10	Forward	5'-AGCCTTATCGGAAATGATCCAGT-3'
	Reverse	5'-GGCCTTGTAGACACCTTGGT-3'
GAPDH	Forward	5'-AGGGCATCTTGGGCTACAC-3'
	Reverse	5'-TGGTCCAGGGTTTCTTACTCC-3'

SOD1, superoxide dismutase 1; CAT, catalase; GPx, glutathione peroxidase; Keap1, Kelch-like ECH-associated protein 1; Nrf-2, nuclear erythroid 2-related factor 2; TLR-4,Toll-like receptor-4;

NF-κB, nuclear factor kappa B; IL-6, interleukin 6; IL-1β, interleukin 1β; IL-10, interleukin 10; TNF-α, tumor necrosis factor -α; GAPDH, glyceraldehyde-3-phosphate dehydrogenase.
